# Blunted Diurnal Cortisol Activity in Healthy Adults with Childhood Adversity

**DOI:** 10.3389/fnhum.2017.00574

**Published:** 2017-11-28

**Authors:** Yuliya I. Kuras, Naomi Assaf, Myriam V. Thoma, Danielle Gianferante, Luke Hanlin, Xuejie Chen, Alexander Fiksdal, Nicolas Rohleder

**Affiliations:** ^1^Laboratory for Biological Health Psychology, Department of Psychology and Volen National Center for Complex Systems, Brandeis University, Waltham, MA, United States; ^2^Chair of Health Psychology, Department of Psychology, Friedrich-Alexander-Universität Erlangen-Nürnberg, Erlangen, Germany

**Keywords:** childhood adversity, stress, diurnal cortisol, HPA, CAR

## Abstract

Childhood adversity, such as neglect, or physical, emotional, or sexual abuse, is prevalent in the U.S. and worldwide, and connected to an elevated incidence of disease in adulthood. A pathway in this relationship might be altered hypothalamic-pituitary-adrenal (HPA) axis functioning, as a result of differential hippocampal development in early life. A blunted diurnal cortisol slope is a precursor for many disorders. While studies have focused on HPA reactivity in relation to childhood adversity, there has been markedly less research on basal HPA functioning in those with low-to-moderate adversity. Based on previous research, we hypothesized that adults with low-to-moderate childhood adversity would have altered HPA axis functioning, as evidenced by a blunted diurnal cortisol slope and altered cortisol awakening response (CAR). Healthy adults aged 18–65 (*n* = 61 adults; 31 males and 30 females) completed the Childhood Trauma Questionnaire. Participants provided at-home saliva samples on two consecutive days at wake-up, and 30 min, 1, 4, 9, and 13 h later; samples were averaged over the 2 days. We found that low-to-moderate childhood adversity predicted lower morning cortisol (β = -0.34, *p* = 0.007, *R*^2^ = 0.21), as well as a blunted cortisol slope (β = 2.97, *p* = 0.004, *R*^2^ = 0.22), but found no association with CAR (β = 0.19, *p* = 0.14, *R*^2^ = 0.12). Overall, we found that in healthy participants, low-to-moderate adversity in childhood is associated with altered basal HPA activity in adulthood. Our findings indicate that even low levels of childhood adversity may predispose individuals to disease associated with HPA dysregulation in later life.

## Introduction

Childhood adversity is a broad term that encompasses many negative experiences prior to adulthood, ranging from unpleasant to traumatic. These experiences may include physical, emotional, or sexual abuse, neglect, separation from parent, parental loss, or instances of domestic or community violence. The World Health Organization estimates that, across countries with widely varying levels of economic development, roughly 40% of children experience at least one adverse event; of those, children have a 60% chance of experiencing a second adverse event (Kessler et al., 2010). Adverse experiences tend to co-occur and persist over time. Though staggering, this figure has been similarly reported in the United States by the most recent National Survey of U.S. Children’s Health (Child Health Initiative, 2011).

Research has connected childhood adversity to an elevated incidence of negative health outcomes in adulthood. Adverse experiences in childhood are associated with adult deficiencies in cardiovascular and immune health, metabolic control, learning, behavioral and psychological functioning, increased propensity toward unhealthy lifestyles, and in the increased prevalence of chronic illnesses (Shonkoff et al., 2012). These illnesses include adult asthma (Bhan et al., 2014), chronic pain (Sweeney et al., 2015), and life-threatening disorders such as chronic obstructive pulmonary disease (Anda et al., 1999), ischemic heart disease, cancer, skeletal fractures, liver disorders (Felitti et al., 1998; Dong et al., 2003), strokes, and diabetes (Gilbert et al., 2015).

One theorized pathway in the connection between childhood adversity and adult health is through altered hypothalamic-pituitary-adrenal (HPA) axis feedback-loop functioning. This is thought to happen as a result of biological programming, which occurs during sensitive periods in development, and calibrates the function of stress systems. Research suggests that this programming happens exceedingly early in life, even before birth. Prenatal exposure to smoking (Stroud et al., 2014), environmental exposures (Swanson et al., 2009), maternal stress (Entringer et al., 2008), and subpar nutrition (McMillen et al., 2008) have been found to alter HPA axis functioning in offspring. Early childhood is a critical time for the development of the hippocampus, and stress may impact the trajectory of this development by way of glucocorticoid regulation of gene expression in the hippocampus (Lupien et al., 2009). Adverse conditions can change the functioning and size of the hippocampus, altering regulation of HPA axis activity. Indeed, studies have linked childhood adversity to reduced hippocampal volume in cases of physical and sexual abuse (Andersen and Teicher, 2009). Glucocorticoid receptors (GRs) are important regulators of HPA feedback within the hippocampus, as changes in GR activity impact stress response. Prenatal (Oberlander et al., 2008) and childhood adversity (Romens et al., 2015) are associated with changes in GR methylation and transcription. In this way, childhood adversity may lead to reduced HPA axis feedback sensitivity, resulting in altered HPA basal activity.

Altered HPA axis reactivity to stress has been observed in individuals reporting childhood adversity, but studies have found both hypo- and hyper-reactivity in cortisol responses to stress (Tarullo and Gunnar, 2006; Engert et al., 2010). These opposing patterns complicate the interpretation of the role of childhood adversity in altered HPA axis reactivity, and might stem from confounding factors, such as psychopathology. In previous stress reactivity research, it has been found that those with a history of both childhood abuse and symptoms of anxiety and depression had a hyper-reactive HPA response, compared to those with childhood abuse but no symptomology; further, those with a history of childhood abuse and a diagnosis of Major Depression exhibited the highest HPA response of these groups (Heim et al., 2000).

While previous studies have addressed childhood adversity and HPA axis stress reactivity, there has been markedly less research on basal HPA axis function. The HPA axis, controlled by the paraventricular nucleus of the hypothalamus, has a circadian rhythm of hormone secretion. This pattern is seen in cortisol, which is at its highest 30-min after waking in the morning, and decreases throughout the day (Brandenberger et al., 1984). A flat diurnal cortisol slope is considered maladaptive, and a precursor of disorders that result from the increased inflammation associated with dysregulated HPA patterns (Nijm et al., 2007). These include chronic fatigue syndrome, fibromyalgia, chronic headaches, and rheumatoid arthritis (Heim et al., 2000). Altered diurnal HPA function has also been implicated in early lung and breast cancer death (Sephton et al., 2000, 2013). Therefore, basal HPA activity may connect childhood adversity to adult health.

Research on basal cortisol patterns in those with childhood adversity has primarily focused on children (for a review see: Tarullo and Gunnar, 2006). In children with severe levels of maltreatment, studies have found elevated cortisol levels throughout the day (Cicchetti and Rogosch, 2001; Carrion et al., 2002), while studies of orphanage- and foster-reared children with adversity have found that cortisol levels are lower in the morning, and the overall diurnal cortisol slope is flatter (Kaufman, 1991; Dozier et al., 2006). In terms of the cortisol awakening response (CAR), the rise in cortisol within 30 min of waking, it has been found that infants experience a higher CAR response when exposed to cigarette smoke *in utero*, and those in low-income or high-stress families (Saridjan et al., 2010).

Less is known about diurnal HPA activity in adults with a history of childhood adversity. One study found that adults with childhood adversity and an anxiety disorder exhibited lower cortisol levels throughout the day, and blunted diurnal cortisol slopes, compared to controls with an anxiety disorder but no maltreatment (van der Vegt et al., 2009). Another study found that high levels of childhood adversity related to lower cortisol levels 45 min after waking, but elevated cortisol 3 h later, resulting in flatter slopes (Power et al., 2012). Further, in a sample of Anorexia and Bulimia Nervosa patients, those with childhood trauma had blunted CAR (Monteleone et al., 2015). Alternatively, one study found higher CAR in adults with low care in childhood (Engert et al., 2011), but also increased cortisol levels in the evening. This CAR increase has also been found in postpartum women with severe childhood adversity (Gonzalez et al., 2009). In addition, elevated diurnal cortisol levels were found in women with chronic pain and severe childhood trauma (Nicolson et al., 2010).

The current literature on childhood adversity and altered diurnal HPA activity has focused either on children, or on adults with severe levels of childhood adversity or comorbidity. To add complexity, in examining childhood adversity, the majority of diurnal HPA research has been conducted on children, while the majority of stress reactivity HPA research has been conducted on adults (Tarullo and Gunnar, 2006). In order to add to the existing literature, we set out to test the association between low-to-moderate levels of childhood adversity and HPA axis functioning in healthy adults. Based on previous findings, we hypothesized that healthy adults with low-to-moderate childhood adversity would have dampened HPA axis functioning, with a flatter diurnal cortisol slope and an altered CAR.

## Materials and Methods

### Sample

Adults (age 18–65 years) were recruited from Brandeis University and the surrounding towns via newspaper and magazine advertisements. Participants were *n* = 61 healthy adults (31 males; 30 females), with a mean age of 33.8 years (*SD* = 2.3), a mean body fat of 25.3% (*SD* = 7.1), and a mean BMI of 25.3 kg/m^2^ (*SD* = 3.6). Participants underwent a medical and psychological screening by telephone before testing and were invited to participate only if they met following selection criteria: (a) body mass index (BMI) between 18 and 35 kg/m^2^; (b) for females: luteal phase of menstrual cycle at time of participation, defined as the 2-week period leading up a female participant’s next expected cycle start date; (c) absence of psychiatric, endocrine, or cardiovascular diseases, or other specific chronic diseases; (d) no intake of psychoactive drugs, beta-blockers, gonadal steroids (hormonal contraceptives), hormone replacement therapy in menopausal women, GCs; (e) non-smoker; and (f) not under any extraordinary stress at the current time, such as bereavement, the ending of a relationship, or great difficulty with school or work. Individuals were paid for their participation. Weight and body fat measurements were taken using a Seca Supra Plus 720 column scale (Seca, Hamburg, Germany), via a bioelectrical impedance analysis.

### Procedure

Participants were asked to come to the laboratory at Brandeis University. Written informed consent was obtained prior to participation. Participants filled out questionnaires regarding their early life experiences and demographic information. Participants were given Salivette collection devices (Sarstedt, Newton, NC, United States) to take home, as well as instructions for accurate use and timing for this study. Participants were asked to provide 12 saliva samples while conducting their average daily routine over the course of the two following days. Samples were taken at wake-up time, wake-up + 30 min, wake-up + 1 h, wake-up + 4 h, wake-up + 9 h, and finally wake-up + 13 h, on two consecutive days. Participants kept written diary recordings of the exact time each sample was collected. We considered sampling times as compliant when participants collected the wake-up and wake-up + 30 min samples within 10 min of the expected time, the wake-up + 1 h, wake-up + 4 h, and wake-up+9 h samples within an hour of the expected time, and the wake-up + 13 h sample within 2 h of the expected time, as consistent with previous literature (Hoyt et al., 2016; Stalder et al., 2016; Valentino et al., 2017). While several participants had a non-compliant sample time on 1 day (eight participants in the control sample; seven participants in the childhood adversity sample), no participants reported a non-compliant sample time for the same sample on both days; therefore, no participants needed to be excluded from the averaged calculation. Diaries of 15 participants could not be used. In the interest of not reducing our sample, and because the majority of participants had diaries, all of which were found to be compliant with our sampling protocol, we did not exclude these participants based on unusable diaries. Upon return of the samples, salivettes were centrifuged at 2000 *g* for 5 min, and saliva was stored at -20°C until processing. This study was carried out in accordance with the recommendations of Human Subjects in Research, Full Committee Review, with written informed consent from all subjects. All subjects gave written informed consent in accordance with the Declaration of Helsinki. All procedures were approved by the Brandeis University Institutional Review Board approved all procedures.

### Measures

Childhood adversity was assessed using the Childhood Trauma Questionnaire (CTQ, Fink et al., 1995), a 28-item, five point likert-scale measure. The CTQ is a widely used tool to assess childhood adversity, and has been cited in related research more than 1,000 times (MacDonald et al., 2016). The CTQ asks respondents to report on childhood experience across five types of childhood maltreatment; physical abuse (“I got hit so hard by someone in my family that I had to see a doctor or go to the hospital”), sexual abuse (“Someone tried to touch me in a sexual way, or tried to make me touch them”), emotional abuse (“People in my family called me things like “stupid,” “lazy,” or “ugly”), physical neglect (“I had to wear dirty clothes”), and emotional neglect (“My parents were too drunk or high to take care of the family”). The CTQ yields a sum score of overall adversity, out of a possible range of 25–125, and a score in each of the subscales, ranging from 5 to 25. This scale showed good reliability (α = 0.81) in this sample. Scores were used to split the sample into two groups, those with “low-to-moderate” childhood adversity and those with below threshold reported history of adversity, labeled as the “no adversity” group (see below).

### Measurement of Diurnal Cortisol

Salivary free cortisol levels were measured in our laboratory at Brandeis University using a competitive chemiluminescence immunoassay (CLIA; IBL-International, Toronto, ON, Canada). Inter- and intra-assay coefficients of variation were below 10%.

### Statistical Analyses

All statistical analyses were performed using SPSS (24) software packages (IBM, Chicago, IL, United States). Cortisol at each time point was averaged across the 2 days in order to increase the reliability of the measurements. CAR was derived from the area under the curve with respect to ground (AUCg) of the first three (morning) time points {Pruessner et al., 2003}. Diurnal cortisol slope was calculated from the decline of cortisol during the day, without including the CAR. To address the hypothesis that individuals with childhood adversity have altered HPA axis activity, we used two approaches: first, we used hierarchical linear regression to test the continuous relation between total CTQ score and cortisol output at wake-up and bedtime, as well as with the CAR, and diurnal cortisol slope. Second, to examine differences between those with and without childhood adversity, two groups were formed based on self-reported childhood adversity; one group with participants who met criteria for childhood adversity, and one group with participants who did not. This division was based on previously established cutoff scores for CTQ and each subscale. The cut off scores for physical abuse, physical neglect, and sexual abuse were 8. The cut off score for emotional neglect was 15, and emotional abuse was 10. The total CTQ cut off score was 41 (Walker et al., 1999). Analysis of covariance (ANCOVA) was used to test for differences in wake-up and bedtime cortisol levels, CAR, and diurnal cortisol slope. Because HPA axis function is influenced by age, sex, and body fat (McInnis et al., 2015), we controlled for these variables in all analyses. Additionally, a repeated measures ANCOVA, including all of the cortisol time-points, other than CAR, was used to examine diurnal cortisol slope.

## Results

### Childhood Adversity Self-Report Data

Participants had overall CTQ sum scores ranging from 37 to 73 (mean = 45.4 ± 8.7 SD). Twenty-nine participants scored in the “low-to-moderate childhood adversity” range, and 32 had no childhood adversity, based on previously established cut-offs (Walker et al., 1999). For each subscale, the range of scores for the responses, as well as the means were as follows: physical abuse 5–20 (mean = 6.6 ± 3.1 SD), emotional abuse 5–19 (mean = 6.6 ± 3.1 SD), physical neglect 5–15 (mean = 6.3 ± 2.2 SD), and emotional neglect 5–19 (mean = 9.8 ± 4.0 SD). Scores for childhood experiences of sexual abuse were particularly low, ranging from 5 to 6 (mean = 5.1 ± 2.6 SD). See **Table 1** for descriptive statistics of CTQ results. We found a non-significant, but trend-level sex difference for childhood physical neglect score (*p* = 0.096), with men scoring higher, and no additional no sex differences among the remaining CTQ subscales, or the composite score (all *p*’s > 0.178).

**Table 1:** Childhood Trauma Questionnaire Scores in overall childhood adversity, CTQ total, and each of the five subscales, for all participants.

CTQ scale	Mean (*SD*)	Range
CTQ total	45.4 (8.7)	37–73
Physical abuse	6.6 (3.1)	5–20
Emotional abuse	8.8 (3.7)	5–19
Sexual abuse	5.1 (2.6)	5–6
Physical neglect	6.3 (2.2)	5–15
Emotional neglect	9.8 (4.0)	5–19

### Diurnal Cortisol

The diurnal cortisol curve reflected an expected pattern, based on previous research, with a significant time effect (*F*_(2.7,166.8)_ = 72.9; *p* < 0.001). Morning cortisol levels peaked at the expected CAR time point, 30 min after wake-up, and gradually declined until the last time point, 13 h post wake-up. We found a marginally significant sex difference at 1 h post wake-up (*p* = 0.05), indicating that women had higher cortisol output at this time-point, but no other sex differences for any of the cortisol time points (all *p*’s > 0.11).

### Childhood Adversity and Cortisol at Wake-Up and Bedtime

**FIGURE 1:**
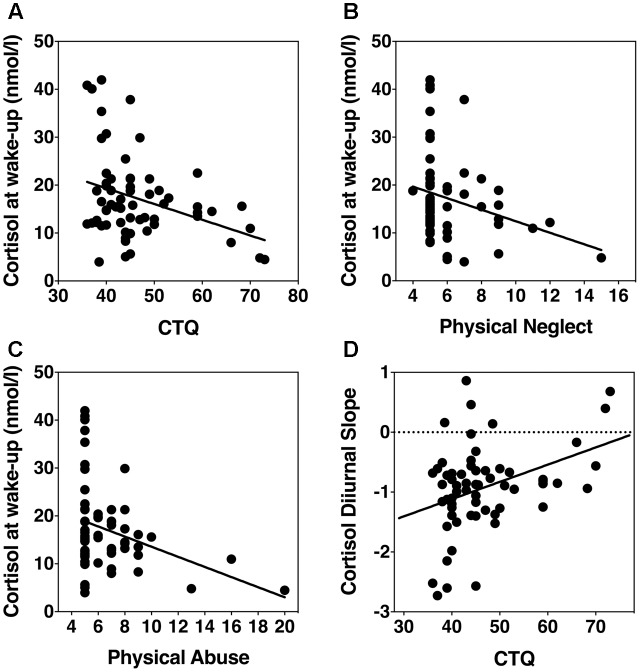
Scatterplots showing associations of childhood adversity with HPA axis basal activity. **(A)** Overall CTQ score and wake-up cortisol levels. **(B)** Physical neglect score and wake-up cortisol levels. **(C)** Physical abuse and wake-up cortisol levels. **(D)** CTQ and diurnal cortisol slope.

Linear regression was used to test the continuous relation between total CTQ score and cortisol output at wake-up and bedtime. Controlling for age, sex, and body fat, we found that overall CTQ score was the strongest predictor of cortisol at wake-up (β = -0.34, *p* = 0.007, *R*^2^ = 0.21); but not at bedtime (β = 0.11, *p* = 0.41, *R*^2^ = 0.11) (**Figure 1A**). Using the CTQ subscales in our regression models, we found that physical neglect (β = -0.32, *p* = 0.002, *R*^2^ = 0.24) and physical abuse (β = -0.34, *p* = 0.006, *R*^2^ = 0.21) were predictive of cortisol output at wake-up, controlling for age, sex, and body fat (**Figures 1B,C**). The remaining CTQ subscales were not predictive of cortisol at wake-up, or at bedtime (all β < 0.11, all >*p* = 0.41).

Using ANCOVA, we tested for group differences between those with and without childhood adversity. We found that the childhood adversity group had significantly lower cortisol levels at the wake-up time point (*F*_(1,60)_ = 4.15, *p* = 0.046, η^2^ = 0.07), controlling for age, sex, and body fat. ANCOVA did not reveal a significant difference between those with and without childhood adversity and bedtime cortisol output (*F*_(1,60)_ = 0.09, *p* = 0.76, η^2^ = 0.002).

### Childhood Adversity and Cortisol Awakening Response

In order to examine the continuous relation between childhood adversity and CAR, hierarchical linear regression was used. Controlling for age, sex, and body fat, we found no significant relation between CAR and total CTQ score (β = 0.19, *p* = 0.14, *R*^2^ = 0.12), or any of the subscales (all β < 0.05, all *p* > 0.67, *R*^2^ = 0.02).

Using ANCOVA, we tested for group differences between those with and without a history of childhood adversity. We found no significant relation between CTQ score and CAR (*F*_(1,60)_ = 2.68, *p* = 0.11, η^2^ = 0.01), or any of the subscales with CAR (all *F* > 0.84, all *p* > 0.36), controlling for age, sex, and body fat.

### Childhood Adversity and Diurnal Cortisol Slope

Next, we analyzed the association between diurnal cortisol slope and childhood adversity as a continuous variable. Controlling for age, sex, and body fat, a linear regression revealed that overall CTQ score significantly predicted diurnal cortisol slope (β = 2.97, *p* = 0.004, *R*^2^ = 0.22) (**Figure 1D**). Diurnal cortisol slope was also predicted by physical neglect (β = 2.7, *p* = 0.009, *R*^2^ = 0.20), physical abuse (β = 3.37, *p* = 0.001, *R*^2^ = 0.25), emotional abuse (β = 2.5, *p* = 0.02, *R*^2^ = 0.18), but not emotional neglect (β = 0.11, *p* = 0.4, *R*^2^ = 0.10), or sexual abuse (β = 0.022, *p* = 0.87, *R*^2^ = 0.09).

**FIGURE 2:**
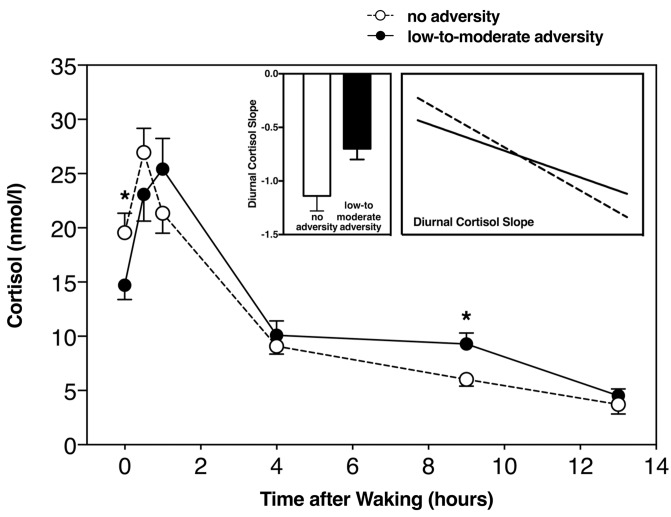
Diurnal cortisol slope including all time-points in those with and without childhood adversity, with slope score difference between the two groups (left insert), and diurnal slope without CAR (right insert). ^∗^Significant at *p* < 0.05.

In order to test for group differences between those with and without childhood adversity, we used an ANCOVA. Controlling for age, sex, and body fat, we found that those with a history of childhood adversity had a less negative slope (*F*_(1,60)_ = 5.3, *p* = 0.025, η^2^ = 0.07) (**Figure 2**). In testing for group differences within each subscale, we found a significant difference in diurnal cortisol slope in those with a history of childhood physical neglect (*F*_(1,60)_ = 6.6, *p* = 0.01, η^2^ = 0.11), and a non-significant, but trend-level difference in those with emotional abuse (*F*_(1,60)_ = 3.4, *p* = 0.07, η^2^ = 0.06), but not any other subscale (all *F* > 2.5, all *p* > 0.12).

We used a repeated measures ANCOVA, including average cortisol at each of the saliva collection time points listed above. Controlling for age, sex, and body fat, the repeated measures ANCOVA revealed significant differences between those with and without childhood adversity and diurnal cortisol slope (*F*_(1,60)_ = 4.29, *p* = 0.009, η^2^ = 0.19). Repeated measures ANCOVA revealed a significant difference in diurnal cortisol slope between those with and without physical neglect (*F*_(1,60)_ = 6.8, *p* = 0.01, η^2^ = 0.10), but we did not find this to be the case with any of the other subscales (all *F* > 2.1, all *p* > 0.11). It can be observed that those with low-to-moderate childhood adversity had a less steep, or blunted diurnal cortisol slope, in line with our previous analyses (**Figure 2**).

## Discussion

Overall, we found a relation between low-to-moderate childhood adversity and altered diurnal HPA axis activity in healthy adults. Specifically, we found that those with a history of childhood adversity had lower cortisol levels upon waking, and a blunted diurnal cortisol slope. In addition, we found that a history of physical neglect and physical abuse were predictive of lower cortisol at wake-up, and that physical neglect, physical abuse, and emotional abuse were predictive of a flatter diurnal cortisol slope. We did not find any significant relation between childhood adversity and CAR.

Our results are in line with those previously found in research conducted on children, including lower cortisol levels in the morning, and overall blunted diurnal cortisol slopes (Kaufman, 1991; Dozier et al., 2006). In contrast, our findings contradict other studies of children that found that those with severe adversity had elevated levels of cortisol throughout the entire day (Cicchetti and Rogosch, 2001; Carrion et al., 2002). This may be due to varying levels of adversity in participants among the study samples. Further, although the participants in our study had low-to-moderate levels of childhood adversity, our results are in line with several previous studies focusing on adults with a history of childhood adversity and either comorbid conditions, or severe maltreatment, which have demonstrated that childhood adversity is associated with decreased morning cortisol levels (Power et al., 2012), and blunted diurnal cortisol slopes (van der Vegt et al., 2009). However, our findings of a blunted diurnal cortisol slope contradict the results of one previous research indicating higher overall diurnal cortisol levels in those with severe childhood trauma (Nicolson et al., 2010). This study extends upon the previous literature by demonstrating that even low-to-moderate levels of childhood adversity are related to flatter diurnal slopes in otherwise healthy adults.

Prior studies have found a relation between the CAR and childhood adversity in both studies of children and adults. Studies of children who have experienced childhood adversity have found elevated CAR (Saridjan et al., 2010), while studies on adults have found both exaggerated (Gonzalez et al., 2009; Monteleone et al., 2015) and blunted (Engert et al., 2011) CAR. We were not able to provide further evidence for either outcome, as we did not find any relation between adversity history and CAR. This may be due to the difference in levels of childhood adversity between those in our study and the samples in previous work, potentially suggesting different trajectories among those with low-to-moderate levels of adversity and those with severe levels. Alternatively, it is possible that the duration of exposure to adversity, or the amount of time from onset of adversity may play a role in HPA basal activity. Adults that have experienced childhood adversity have lived with this experience for longer than children, who may still be experiencing adversity or just recovering; therefore, differential basal HPA activity patterns between children and adults may be expected. This would be consistent with findings of a meta-analysis of chronic and traumatic stress and their associations with HPA axis activity, in which longer duration of stress, or longer time since trauma were predictive of lower HPA axis activity, which would also include flatter diurnal slopes (Miller and Chen, 2007).

Changes in HPA feedback loop functioning are a potential mechanism driving our findings. Adults who have experienced low-to-moderate adversity in childhood may have altered programming of hippocampal feedback regulation, leading to deviations in stress response and basal HPA functioning. Due to the time-sensitive nature of hippocampal development, early life adversity may play a large role in establishing the parameters of glucocorticoid regulation of gene expression in this structure (Lupien et al., 2009). Since early life adversity has the potential to modify GR methylation and transcription, adversity during childhood may lead to reduced HPA axis feedback sensitivity (Romens et al., 2015), resulting in the altered HPA axis basal activity that we find in our study.

Our findings of altered basal HPA axis activity suggest that even in the presence of low-to-moderate levels of childhood adversity, there may be a predisposition for disease. It is likely that due to this basal HPA axis activity dysregulation and potential altered hippocampal functioning, adults with an adverse childhood background are at an increased risk for disease. The blunted diurnal cortisol slope seen in this sample of otherwise healthy adults may be viewed as a precursor of disorders that result from increased inflammation (Nijm et al., 2007), such as immune and metabolic disease (Heim et al., 2000; Shonkoff et al., 2012), and disorders associated with coronary health (Felitti et al., 1998; Dong et al., 2003).

This study had several limitations. Participants in the study were instructed to collect saliva at home, without supervision. It is possible that there was some level of non-compliance with instructions. Although we averaged 2 days of collection for each time point in order to counteract this possibility, this study did not employ electronic monitoring of saliva collection; future work should reduce this potential for error. In addition, our low-to-moderate adversity group reported a particularly low incidence of sexual abuse. Self-report data is subject to recall bias and other psychosocial factors; it is possible that participants misreported information, or that our sample is not representative of all types of low-to-moderate childhood adversity. Further, participants had varying types of adverse events, duration of events, and onset age of events. Future studies should work to address these specific factors in their connection to altered HPA functioning. Finally, our sample had a large age range; while we statistically controlled for this, older adults have had a longer time-course from onset of childhood adversity, which may play a role in the perception of the adverse events, as well as in physiological alterations leading from the events.

Taken together, we found that a history of low-to-moderate early life adversity is associated with lower morning cortisol levels, and a flatter diurnal cortisol slope. In general, it is possible that factors such as onset age of trauma and compounding stressful events in adulthood may differentially impact patterns of activity in the HPA axis, leading to hypo- versus hyper-activity of the HPA. Future work should address childhood adversity and HPA axis function at multiple time points, in order to understand whether HPA axis activity patterns change over time, with special attention to how each pattern relates to overall health. The results of this study, which indicate that diurnal HPA axis activity alterations can be seen in those with even low-to-moderate levels of adversity are meaningful because HPA axis dysregulation is considered a risk factor for many disorders. These results point to low-to-moderate childhood adversity as a risk factor for disease associated with a dysregulated HPA axis.

## Author Contributions

NR was responsible for study design. YK, NA, MT, DG, LH, XC, and AF were responsible for participant testing. YK, NA, and NR conducted data analysis. All authors contributed to and have approved the final version of the manuscript.

## Conflict of Interest Statement

The authors declare that the research was conducted in the absence of any commercial or financial relationships that could be construed as a potential conflict of interest.
